# Turkish Adaptation of the Clean Food Consumerism Scale: A Validity and Reliability Study

**DOI:** 10.3390/nu18193274

**Published:** 2026-10-05

**Authors:** Simge Sipahi Uygur, Nihan Çakır Biçer, Ece Öneş, Sena Roberts, Selen Köksal, Perim Fatma Türker, Murat Baş

**Affiliations:** 1 Department of Nutrition and Dietetics, Faculty of Health Sciences, Acibadem Mehmet Ali Aydinlar University, İstanbul 34752, Türkiye; simge.sipahi@acibadem.edu.tr (S.S.U.); ece.ones@acibadem.edu.tr (E.Ö.); sena.roberts@acibadem.edu.tr (S.R.); perim.turker@acibadem.edu.tr (P.F.T.); murat.bas@acibadem.edu.tr (M.B.); 2 Department of Molecular Gastroenterology and Hepatology, Institute of Gastroenterology and Hepatology, Kocaeli University, Kocaeli 41001, Türkiye; 3 Department of Nutrition and Dietetics, Graduate School of Health Sciences, Acibadem Mehmet Ali Aydinlar University, İstanbul 34752, Türkiye; 4 Department of Culinary, Vocational School, Acibadem Mehmet Ali Aydinlar University, İstanbul 34752, Türkiye; selen.koksal@acibadem.edu.tr

**Keywords:** clean food consumerism, scale adaptation, validity, reliability, psychometric evaluation, sustainable nutrition, Turkish adaptation

## Abstract

**Background:** Clean food consumerism reflects consumers’ preferences for foods perceived as natural, authentic, transparent, and health-promoting. This study aimed to culturally adapt the Clean Food Consumerism Scale (CFCS) into Turkish and evaluate its psychometric properties. **Methods:** The CFCS was translated, back-translated, reviewed by experts, pilot-tested, and psychometrically evaluated. For the pilot study and factor analyses, the Turkish version was administered to 1637 adults, who were allocated to three mutually exclusive subsamples: *n* = 180 for the pilot study, *n* = 500 for EFA, and *n* = 957 for CFA. An additional, separate sample of 124 participants completed the CFCS twice at a three-week interval for the test–retest analysis. Internal consistency, construct validity, convergent validity, discriminant validity, criterion validity, and test–retest reliability were assessed using Cronbach’s alpha, item–total correlations, EFA, CFA, composite reliability (CR), average variance extracted (AVE), the Fornell–Larcker criterion, heterotrait–monotrait (HTMT) ratios, correlations with the Sustainable and Healthy Eating Behaviors Scale (SHEBS), and test–retest correlations. **Results:** The 18-item CFCS demonstrated excellent internal consistency (Cronbach’s α = 0.948). Sampling adequacy was supported by the KMO value (0.927) and significant Bartlett’s test (χ^2^ = 7525.229, *p* < 0.001). EFA supported a five-factor structure comprising Perceived Health Benefits, Production Process Transparency, Authenticity, Perceived Ease of Use, and Familiarity, explaining 80.92% of the total variance. CFA supported the five-factor model. After adding one content-justified residual covariance between two closely related Perceived Health Benefits items, the final model showed acceptable fit (χ^2^ = 641.39, df = 124, *p* < 0.001; CFI = 0.965, TLI = 0.956, NFI = 0.957, RMSEA = 0.066 [90% CI 0.061–0.071], SRMR = 0.047, GFI = 0.926, AGFI = 0.899). Standardized factor loadings ranged from 0.748 to 0.938, composite reliability from 0.873 to 0.940, and average variance extracted from 0.697 to 0.771. HTMT ratios for all factor pairs remained below 0.685 and the Fornell–Larcker criterion was satisfied for every factor, supporting discriminant validity. Test–retest reliability over three weeks was excellent (r = 0.889, *p* < 0.001), and CFCS scores were positively correlated with SHEBS scores (r = 0.441, *p* < 0.001). **Conclusions:** The Turkish version of the CFCS demonstrated strong validity, reliability, and temporal stability while preserving the original five-factor structure. It provides a psychometrically sound instrument for assessing clean food consumerism among Turkish-speaking adults and may support future research in nutrition, public health, sustainability, and consumer behavior.

## 1. Introduction

Adopting a healthy dietary pattern throughout one’s lifetime contributes to the prevention of malnutrition and noncommunicable diseases. However, the increase in production of processed food, rapid urbanization, and shifting lifestyles have caused increased consumption of energy-, fat-, sugar-, and salt-rich foods, while reducing the intake of fiber-rich foods, including fruits, vegetables, and whole grains, ultimately leading to significant changes in dietary patterns [1]. Such dietary habits pose adverse consequences for human and environmental health, as the sustainability of ecosystems holds substantial implications for both individual and public health outcomes [2]. In this regard, the Food and Agriculture Organization (FAO) underscores the significance of sustainable diets, defined as “diets with low environmental impacts which contribute to food and nutrition security and to healthy life for present and future generations,” highlighting that food quantity, quality, and diversity should be sufficient and aligned with established nutritional guidelines [2,3].

Nutrition and diet quality are foundational to achieving the 2030 Sustainable Development Goals, with efforts required across production and consumption levels to ensure healthier food access for all [4,5]. The FAO has indicated that one pathway toward food system transformation encompasses strengthening food environments and modifying consumer behavior to promote dietary patterns that exert positive effects on human health and the environment [6]. Therefore, sustainability at a global scale should be prioritized. In this regard, developing educational initiatives and formulating policies by assessing individuals’ levels of awareness, behavior, and attitudes toward sustainability, while simultaneously working to enhance that awareness, are crucial.

In recent times, there has been a growing desire among individuals to acquire information regarding the source, sustainability, composition, and production methods of their food [7]. This consumer-driven movement, referred to as “clean food consumerism,” highlights the significance of transparency, authenticity, and sustainability, while advocating a return to natural additive-free foods and focusing on food production and sourcing [8]. This shift in consumer food preferences has caused an increasing inclination toward foods that are healthier, safer, and composed of recognizable natural ingredients, a trend that has also contributed to an expansion in the market share of products perceived as “clean” [9]. An industry survey conducted by a sales and marketing research organization reported that 81% of shoppers place considerable significance on purchasing clean food products [7]. Beyond individual consumers, the concept of “clean food” has also gained extensive acceptance within the food industry from a production viewpoint. However, the absence of a universally agreed-upon definition of clean food, alongside the lack of a clear and consistent understanding of the concept among several consumers and researchers, and the limited body of scientific literature on the subject, present significant challenges for manufacturers in the development and production of clean-label products [7,8,10]. The Clean Food Consumerism Scale (CFCS), an instrument developed by Hino and Sparks [7], represents the first psychometrically validated instrument designed to operationalize clean food consumerism as a measurable construct. The scale comprises 18 items across five dimensions—perceived health benefits (6 items), perceived ease of use (3 items), familiarity (3 items), authenticity (3 items), and production process transparency (3 items)—each capturing a distinct facet of consumer orientation toward clean food, from outcome expectations and behavioral facilitators to process- and origin-related values. The CFCS was validated across multiple culturally distinct populations and is intended to enable an empirically grounded, comprehensive definition of clean food consumerism [7].

Despite the multi-population validation conducted by Hino and Sparks [7], the cross-cultural applicability of the CFCS beyond the populations included in the original study remains unexamined, as no independent adaptation of the scale into other languages or cultural contexts has been reported in the peer-reviewed literature to date. The adaptation of psychometric instruments for use in new cultural and linguistic contexts requires a systematic process that ensures conceptual, semantic, and experiential equivalence across populations [11], and is particularly consequential when the construct under study—clean food consumerism—is shaped by culturally embedded values such as food authenticity, culinary tradition, and trust in production systems. In Türkiye, consumer interest in natural ingredients, food labeling, and health-oriented food attributes has been documented as a significant and growing trend [12,13], and several food-related scales have been successfully adapted and validated for Turkish populations in recent years [14,15,16]. Nevertheless, no psychometrically validated Turkish instrument currently exists to capture clean food consumerism as a multidimensional construct, limiting the capacity of researchers and practitioners to measure, compare, and respond to this phenomenon in the Turkish context.

The present study therefore aims to culturally adapt the CFCS into Turkish and evaluate its psychometric properties in terms of construct, convergent, discriminant, and criterion validity; internal consistency; and test–retest reliability. By doing so, this study makes two principal contributions to the existing literature: theoretically, it extends the cross-cultural validation of the CFCS beyond its original populations and provides evidence regarding the structural stability of the clean food consumerism construct in a new linguistic and cultural setting; practically, the Turkish CFCS offers a validated measurement tool for researchers, nutrition professionals, and public health policymakers to assess clean food-related consumer attitudes and behaviors in Türkiye, supporting the development of evidence-based dietary communication strategies aligned with national and international sustainability goals.

## 2. Materials and Methods

### 2.1. Study Design and Participants

This study was conducted from 15 November 2025 to 3 March 2026. Data were collected through a self-administered online questionnaire using Google Forms. Participants were recruited through a non-probability snowball sampling approach. The survey link was initially distributed via social media platforms, and participants were asked to share it with other eligible individuals within their social networks. This recruitment strategy was used to obtain an adult sample for the psychometric evaluation of the Turkish version of the CFCS and was not designed to generate a nationally representative sample. Individuals aged 18 years or older who voluntarily agreed to participate in the study were eligible for inclusion. Before accessing the survey, participants were informed about the purpose and procedures of this study and were asked to indicate whether they agreed to participate in this study.

The Acibadem Mehmet Ali Aydinlar University and Acibadem Healthcare Institutions Medical Research Ethics Committee approved the study protocol (approval number: 2025-16/608; approval date: 16 October 2025). All procedures were performed following the ethical principles outlined in the Declaration of Helsinki.

### 2.2. Translation and Cultural Adaptation of the Clean Food Consumerism Scale

For the Turkish adaptation of the CFCS developed by Hino and Sparks [7], permission was obtained from the original authors. The CFCS is an 18-item self-report instrument intended for assessing individuals’ attitudes and behaviors related to clean food consumption [7]. The scale evaluates consumers’ preferences for foods perceived as natural, minimally processed, and free from artificial additives, as well as their purchasing tendencies associated with clean food products. Higher scores denote stronger clean food consumerism tendencies. Three independent translators fluent in English and Turkish translated the CFCS from English to Turkish; the research team compared the resulting translations to develop a standardized Turkish version. Expert dietitians independently evaluated the linguistic clarity and appropriateness of the resulting Turkish version.

The Turkish version of the scale was subsequently translated back into English using the back-translation method; the back-translated text was compared with the original scale. During this process, the statements were evaluated for semantic equivalence, linguistic appropriateness, conceptual consistency, and any phrasing that could cause misunderstandings. The final Turkish version of the scale was developed after making the necessary revisions based on expert opinions.

### 2.3. Sustainable and Healthy Eating Behaviors Scale

Criterion validity was evaluated using the Sustainable and Healthy Eating Behaviors Scale (SHEBS), originally developed by Żakowska-Biemans et al. [3] and later adapted and validated for use in the Turkish population by Köksal et al. [17]. This scale evaluates sustainable and healthy eating behaviors across multiple dimensions. Higher scores indicate greater adherence to sustainable and healthy eating behaviors.

The Turkish version of the SHEBS demonstrated good psychometric properties, with a Cronbach’s alpha coefficient of 0.912 and a test–retest intraclass correlation coefficient of 0.832 [17]. The association between the CFCS and SHEBS scores was examined to assess criterion validity.

### 2.4. Pilot Study

Before the main analyses, a pilot analysis was conducted to assess the scale’s clarity, feasibility, and preliminary reliability. A sample size of at least 5–10 times the number of items recommended in scale development and adaptation studies was considered when determining the sample size for the pilot study. Accordingly, a pilot subsample of 180 participants was used for the 18-item scale (Section 2.5).

The scale used in the pilot study comprised 18 items, which were rated on a five-point Likert scale. Participants were asked to select the option that best suited them for each statement. Item analyses and internal consistency evaluations were conducted on the basis of the collected data.

### 2.5. Sample Size and Power Analysis

Power analysis was employed for determining the sample size for this study. Analyses were performed using R software (v4.3.1) (R Foundation for Statistical Computing, Vienna, Austria), with a significance level of α = 0.05 and a statistical power of 90%. Based on the effect size obtained from the pilot study (r = 0.343), the minimum required sample size for detecting a correlation of this magnitude was approximately 85 participants. The EFA (*n* = 500) and CFA (*n* = 957) subsamples considerably exceeded both this value and the commonly recommended ratio of 10 participants per item (*n* ≥ 180). For the pilot study and factor analyses, an initial pool of 1637 adults was used. Participants were then allocated to three mutually exclusive subsamples using simple random selection in SPSS (v27): *n* = 180 for the pilot study, *n* = 500 for the exploratory factor analysis (EFA), and *n* = 957 for the confirmatory factor analysis (CFA). No participant contributed to more than one of these analyses, so the EFA sample is not a subset of the CFA sample, and the exploratory solution was therefore tested in an independent sample. In addition, a separate sample of 124 participants, who were not included in the pilot, EFA, or CFA subsamples, completed the CFCS twice at a three-week interval for the test–retest analysis. The identifiers confirming that the three subsamples do not overlap are reported in Supplementary File S1.

### 2.6. Psychometric Assessment

The reliability of the scale was assessed using Cronbach’s alpha and corrected item–total correlations. Cronbach’s alpha values of ≥0.70 were considered to indicate acceptable internal consistency. Corrected item–total correlation coefficients of ≥0.30 were considered satisfactory for item discrimination.

The Kaiser–Meyer–Olkin (KMO) measure of sampling adequacy and Bartlett’s test of sphericity were used for investigating the suitability of the scale for factor analysis. KMO values > 0.60 and a statistically significant Bartlett’s test result (*p* < 0.05) indicated suitability for factor analysis. To establish and cross-validate the psychometric properties of the scale, the factor analyses were carried out in two separate, non-overlapping subsamples. First, Exploratory Factor Analysis (EFA) was conducted on a subsample of *n* = 500 participants to uncover the underlying factor structure. Sample adequacy for EFA was verified using the Kaiser–Meyer–Olkin measure (KMO = 0.927), exceeding the threshold of 0.90 that indicates excellent sampling adequacy. Bartlett’s test of sphericity confirmed that the correlation matrix was factorable (χ^2^ = 7525.229, df = 153, *p* < 0.001). Subsequently, Confirmatory Factor Analysis (CFA) was performed on a separate, non-overlapping subsample of *n* = 957 participants to confirm the structural validity and goodness-of-fit of the identified model.

EFA was conducted to investigate the factor structure of the scale. Principal component analysis (PCA) and the Promax rotation method were employed for establishing the factor structure. Factors with eigenvalues > 1.00 were retained, and factor loadings ≥ 0.40 were deemed acceptable. To determine the optimal number of factors in Exploratory Factor Analysis (EFA), both Kaiser’s criterion (eigenvalue > 1.0) and Horn’s Parallel Analysis [18] were employed using the psych package in R (v4.3.1) [19]. Parallel Analysis was executed on 100 Monte Carlo simulated random datasets using the Minimum Residual (minres) extraction method. Components with eigenvalues greater than 1.0 were retained according to Kaiser’s criterion, and this number was supported by the parallel analysis if the corresponding empirical factor eigenvalues exceeded the 95th percentile of the simulated eigenvalues.

CFA was performed on the independent sample (*n* = 957) to validate the factor structure. The model initially specified (Model 1) was a first-order measurement model with five correlated latent factors, no cross-loadings and no correlated residuals. Each of the 18 items loaded on a single factor: Perceived Health Benefits (6 items), Perceived Ease of Use (3 items), Familiarity (3 items), Authenticity (3 items) and Production Process Transparency (3 items). All ten inter-factor covariances were freely estimated. The metric of each latent variable was set by fixing its first indicator loading to 1.0, and standardized solutions are reported throughout. The model was estimated by maximum likelihood, yielding 46 free parameters and 125 degrees of freedom. The final model reported below (Model 2) adds a single residual covariance to this specification and therefore has 47 free parameters and 124 degrees of freedom. Model fit within the CFA framework was evaluated using the chi-square to degrees of freedom ratio (χ^2^/df), Root Mean Square Error of Approximation (RMSEA), Normed Fit Index (NFI), Comparative Fit Index (CFI), Tucker–Lewis Index (TLI), Standardized Root Mean Square Residual (SRMR), Goodness-of-Fit Index (GFI), and Adjusted Goodness-of-Fit Index (AGFI). Model fit was evaluated primarily using RMSEA (≤0.08), SRMR (≤0.08), CFI, TLI and NFI (≥0.90), and GFI and AGFI (≥0.85). RMSEA is reported with its 90% confidence interval, and the interval rather than the point estimate alone was used to judge approximate fit. Because the χ^2^ statistic increases approximately linearly with sample size, χ^2^ and its *p* value are reported for completeness, but the χ^2^/df ratio was not used as a primary criterion [20]. Local sources of misfit were examined using the matrix of standardized residual correlations and modification indices. Where a residual indicated systematic covariation not accounted for by the measurement model, a single modification was considered, and it was adopted only if it was both statistically substantial and interpretable in terms of the content of the items concerned.

Convergent validity was assessed using standardized factor loadings, average variance extracted (AVE), and composite reliability (CR). Convergent validity was considered acceptable when AVE values were ≥0.50 and CR values were ≥0.70. Discriminant validity was assessed using the Fornell–Larcker criterion, in which the square root of the AVE of each factor is compared with its correlations with the remaining factors, and using heterotrait–monotrait (HTMT) ratios evaluated against a threshold of 0.85. The correlations among observed subscale sums, reported in the Results section, do not constitute a test of discriminant validity at the latent level.

The association between scores on the CFCS and SHEBS was investigated to assess criterion validity.

### 2.7. Statistical Analysis

Statistical analyses were performed using SPSS 27.0 (IBM Corp., Armonk, NY, USA) and R software (v4.3.1) (R Foundation for Statistical Computing, Vienna, Austria). Categorical variables were summarized as counts and percentages, whereas continuous variables were summarized as means ± standard deviations, medians, and minimum and maximum values. Normality of the data distribution was evaluated using skewness and kurtosis values alongside the Shapiro–Wilk test. Because formal normality tests such as Shapiro–Wilk are highly sensitive to minor deviations in large sample sizes (*n* > 300), skewness and kurtosis indices were primarily evaluated to assess parametric assumptions. Skewness values ranged from −0.652 to −0.138, and kurtosis values ranged from −0.424 to 0.598, all well within the recommended threshold of [−1.0, +1.0] for univariate normality [21,22]. Consequently, parametric tests, including Pearson correlation analysis, were retained. A *p*-value of <0.05 was considered statistically significant in all analyses.

## 3. Results

### 3.1. Pilot Study

In the pilot study, 33.3% and 66.7% of the participants (*n* = 180) were men and women, respectively. Their mean age was 28.74 ± 10.51 years (Supplementary Table S1). In the pilot study, the Cronbach’s alpha value of the CFCS was 0.950 and the scale was found to have high reliability. According to the results of the item analysis, Cronbach’s alpha-if-item-deleted values for the 18 items ranged from 0.947 to 0.949. Because no corrected item–total correlation was below 0.30, no item needed to be removed, and the CFCS was considered suitable for validity analysis.

### 3.2. Reliability Analysis of the Clean Food Consumerism Scale

Cronbach’s alpha value of the 18-item CFCS was 0.948, and the scale had high reliability (Table 1).

The overall scale had a high Cronbach’s alpha coefficient (α = 0.948), and the corrected item–total correlations ranged from 0.597 to 0.757. All item–total correlations were below the standard threshold of 0.85, suggesting high internal consistency without item redundancy [20]. Moreover, all items contributed substantially to the overall scale reliability. Cronbach’s alpha-if-item-deleted values ranged from 0.944 to 0.947, indicating that removing items from the scale was unnecessary (Table 2).

### 3.3. Validity Analysis of the Clean Food Consumerism Scale

The KMO value of 0.927 indicated excellent sampling adequacy, and Bartlett’s test of sphericity was significant (χ^2^ = 7525.229, df = 153, *p* < 0.001), indicating that the data were suitable for EFA (Table 3).

#### 3.3.1. Exploratory Factor Analysis

##### Descriptive Characteristics of the Participants Included in the Exploratory Factor Analysis (EFA)

Of the 500 individuals participating in the EFA study, 30% and 70% were male and female, respectively. Male participants had a mean age of 30.34 ± 11.63 years. Among them, 59.3% had an associate or bachelor’s degree, 44.7% reported that their income exceeded their expenses, 12.0% had a chronic disease, and their mean body mass index (BMI) was 23.55 ± 1.64 kg/m^2^. Conversely, female participants had a mean age of 31.73 ± 11.94 years. Among them, 59.4% had an associate or bachelor’s degree, 45.7% reported that their income was equal to their expenses, 17.1% had a chronic disease, and their mean BMI was 23.01 ± 4.61 kg/m^2^ (Table 4).

As a result of the EFA, a five-factor structure with factor loadings ≥ 0.40, eigenvalues > 1.0, and explaining 80.922% of the total variance was identified. PCA was conducted to determine the factor structure of the scale; no item with a factor loading < 0.40 was noted. No item showed a difference of less than 0.10 between its primary loading and a cross-loading; therefore, no item was removed from the scale. The resulting factors were labeled as “Perceived Health Benefits,” “Production Process Transparency,” “Authenticity,” “Perceived Ease of Use,” and “Familiarity.” Based on these findings, the Turkish CFCS was confirmed as an 18-item, five-factor scale rated on a 5-point Likert scale, with total scores ranging from 18 to 90 (Table 5).

**Figure 1:**
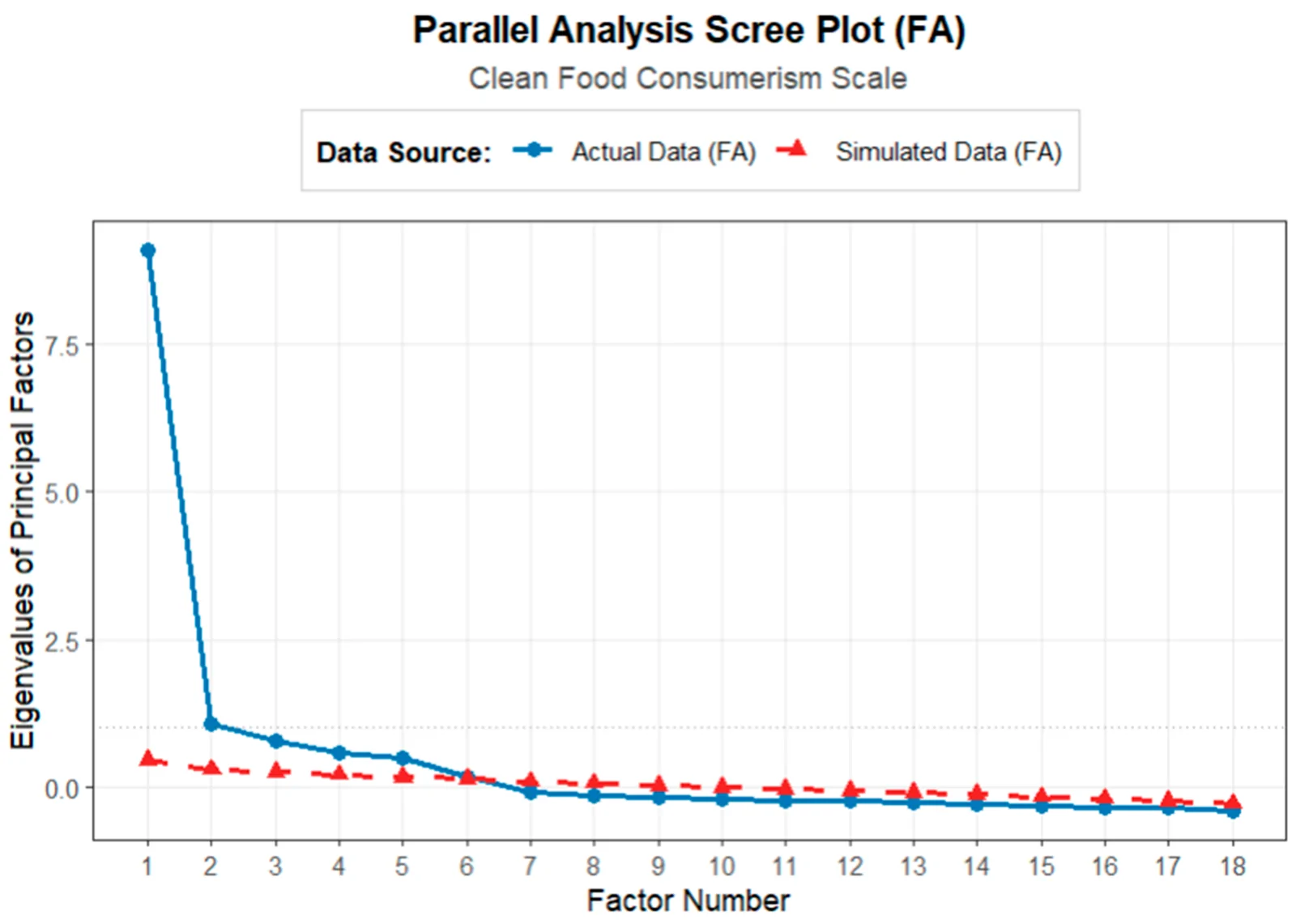
Parallel analysis scree plot comparing empirical eigenvalues with randomly generated Monte Carlo eigenvalues (FA model, 100 iterations).

To determine the number of factors to retain in a rigorous way and avoid the biases of over-factoring associated with using only Kaiser’s criterion (eigenvalues > 1.0), Horn’s Parallel Analysis [18] was performed using the psych package in R (v4.3.1). The empirical eigenvalues from the sample data (*n* = 500) were compared with simulated eigenvalues from 100 Monte Carlo random data matrices. As shown in Figure 1, the empirical factor eigenvalues of the first five factors exceeded the corresponding simulated eigenvalues, whereas from the sixth factor onward the empirical eigenvalues no longer exceeded the simulated values. The parallel analysis therefore supported the five-factor structure identified by Kaiser’s criterion, which explained 80.92% of the total variance and was further cross-validated via Confirmatory Factor Analysis (CFA).

#### 3.3.2. Confirmatory Factor Analysis

Of the 957 individuals participating in the confirmatory factor analysis (CFA) study, 43.3% and 56.7% were male and female, respectively. Male participants had a mean age of 34.57 ± 13.84 years. Among them, 40.6% were married, 48.3% had an associate or bachelor’s degree, 22.9% had a chronic disease, and their mean BMI was 27.99 ± 3.62 kg/m^2^. Conversely, female participants had a mean age of 31.73 ± 12.02 years. Among them, 37% were married, 57.6% had an associate or bachelor’s degree, 23.8% had a chronic disease, and their mean BMI was 24.80 ± 3.93 kg/m^2^ (Table 6).

**Figure 2:**
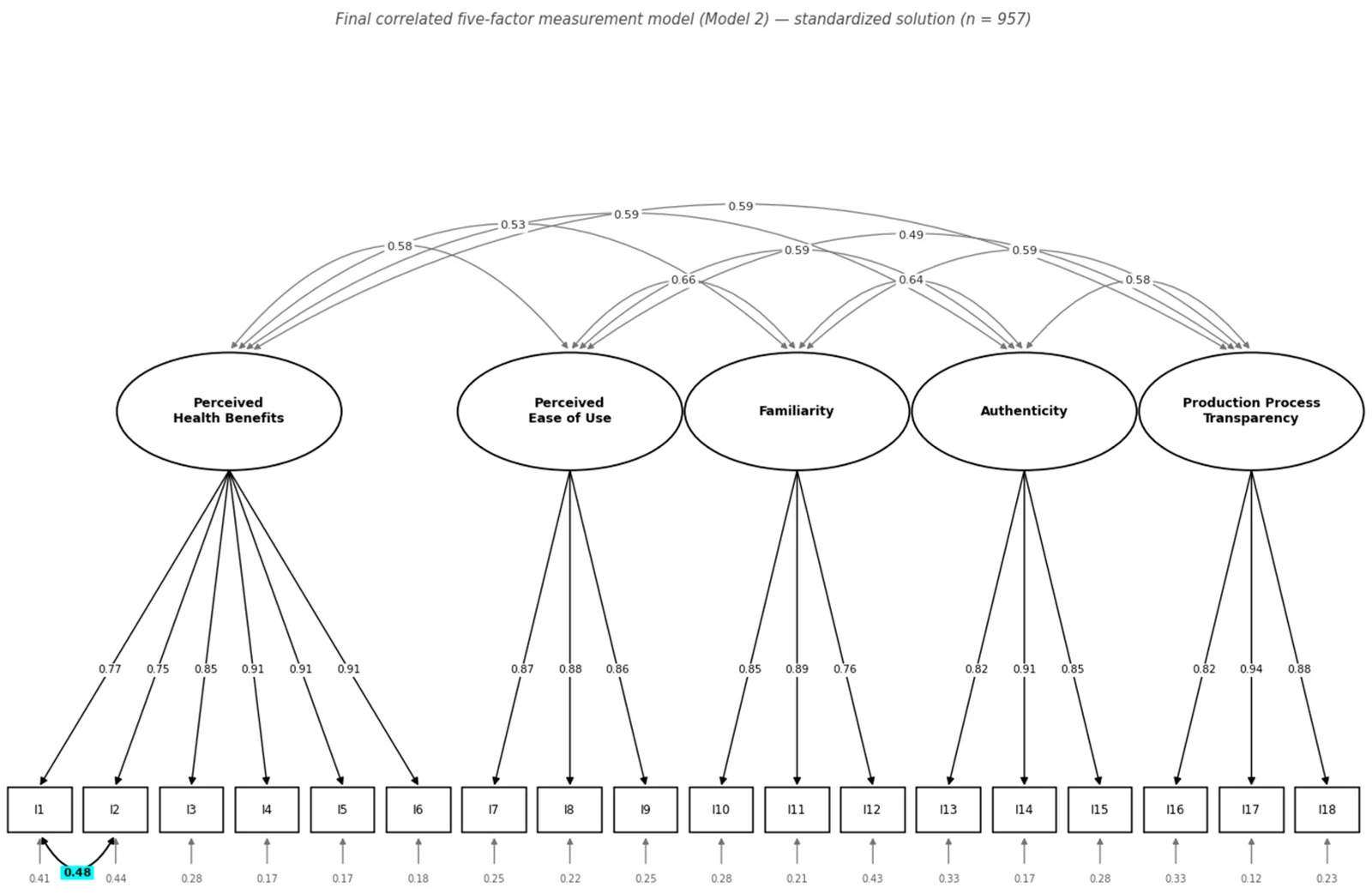
Confirmatory factor analysis of the Turkish Clean Food Consumerism Scale: final correlated five-factor measurement model (Model 2) with standardized parameter estimates (*n* = 957). Values on straight arrows are standardized factor loadings, values on curved double-headed arrows are latent factor correlations, and values below the item boxes are standardized residual variances. The curved arrow linking items 1 and 2 is the residual covariance included in Model 2. Its value (0.48) is the standardized residual correlation between the two items.

CFA was performed for validating the five-factor structure of the CFCS identified through EFA. All standardized factor loadings were statistically significant (*p* < 0.001) and ranged from 0.748 to 0.938, exceeding the recommended threshold of 0.50; no items were removed from the scale (Figure 2). 

The initially specified model (Model 1) yielded χ^2^ = 865.76 (df = 125, *p* < 0.001), CFI = 0.949, TLI = 0.938, NFI = 0.942, RMSEA = 0.079 (90% CI 0.074–0.084), SRMR = 0.045, GFI = 0.896 and AGFI = 0.858. The χ^2^/df ratio was 6.93; because the χ^2^ statistic increases approximately linearly with sample size, this ratio is reported for completeness but was not used as a primary criterion. The incremental and residual-based indices met the recommended thresholds, but the RMSEA point estimate of 0.079 fell only marginally below the 0.08 boundary and the upper limit of its 90% confidence interval (0.084) exceeded that boundary. The data were therefore not sufficient to exclude mediocre approximate fit for Model 1, and local sources of misfit were examined.

Inspection of the standardized residual correlations (Supplementary File S1) showed that the largest residual was between items 1 and 2 of the Perceived Health Benefits subscale (r = 0.164, z = 5.07). Residuals of almost the same size were observed between item 12 (Familiarity) and items 1 and 2 (r = 0.160 and 0.157), and item 12 was involved in seven of the ten largest residuals, including those with all three Authenticity items (r = 0.110–0.130). Two sources of local misfit were therefore identified. Modification indices (Supplementary File S1) pointed to the same two sources: the largest index concerned the residual covariance between items 1 and 2 (MI = 212.1), followed by a cross-loading of item 12 on the Authenticity factor (MI = 91.8). Items 1 and 2 are the only items in the Perceived Health Benefits subscale that express a general evaluation of the health and nutritional value of clean food (“Clean food contains a lot of vitamins and minerals” and “Clean food is healthier compared to other foods”), whereas items 3 to 6 refer to naturalness and production-related attributes (resemblance to organic food, no genetic modification, no additives and natural ingredients only). This shared content therefore provides a substantive reason to expect covariation beyond the common factor. Item 12 (“Clean food reminds me of what I used to eat during my childhood”) refers to tradition and personal food history, which overlaps conceptually with the content of the Authenticity items (authentic, traditional, and associated with one’s ethnic background); this is consistent with its secondary loadings on the Authenticity and Perceived Ease of Use factors in the EFA (Table 5).

Adding a cross-loading or residual covariances for item 12 would have altered the simple structure of the original instrument and compromised comparability with the original scale; in line with the planned restriction to a single, content-justified modification, only the residual covariance between items 1 and 2 was therefore added (Model 2), and the residuals involving item 12 were not modelled. This modification produced a significant improvement in fit (Δχ^2^ = 224.37, Δdf = 1, *p* < 0.001) and yielded χ^2^ = 641.39 (df = 124, *p* < 0.001), CFI = 0.965, TLI = 0.956, NFI = 0.957, RMSEA = 0.066 (90% CI 0.061–0.071), SRMR = 0.047, GFI = 0.926 and AGFI = 0.899. The upper limit of the RMSEA confidence interval fell below the 0.08 boundary. The residual correlation between items 1 and 2 was 0.48 (unstandardized covariance of 0.297, SE = 0.024, *p* < 0.001; 0.206 when the covariance is expressed in the metric of the observed item variances), indicating substantial shared variance beyond the common factor. Factor loadings, composite reliability, average variance extracted and the latent factor correlations were essentially unchanged between the two models, indicating that the modification affected local fit rather than the substantive interpretation of the measurement structure. Model 2 was therefore retained as the final measurement model. Fit statistics for both models are presented in Table 7. The parameter estimates reported in Table 8 and Figure 2 are those of Model 2. Standardized loadings, composite reliability and average variance extracted for each factor are presented in Table 8, and discriminant validity results in Table 9. Full parameter estimates, including standard errors and latent factor correlations, are provided in Supplementary Table S2.

#### 3.3.3. Mean and Median Values of the CFCS

The total score of the scale ranged from 18 to 90, and the mean was 59.92 ± 14.71. The mean score of the “Perceived Health Benefits” subscale was 20.49 ± 6.33, and the subscale scores ranged from 6 to 30. The mean score of the “Production Process Transparency” subscale was 10.38 ± 3.11, and the subscale scores ranged from 3 to 15. The mean score of the “Authenticity” subscale was 9.55 ± 2.93, and the subscale scores ranged from 3 to 15. The mean score of the “Perceived Ease of Use” subscale was 9.44 ± 2.94, and the subscale scores ranged from 3 to 15. The mean score of the “Familiarity” subscale was 10.05 ± 2.91, and the subscale scores ranged from 3 to 15. Additionally, the subscale and total scores demonstrated acceptable levels of skewness (ranging from −0.652 to −0.138) and kurtosis (ranging from −0.424 to 0.598), affirming the univariate normality of the scores (Table 10).

#### 3.3.4. Test–Retest Reliability Findings of the CFCS

Test–retest reliability was assessed in a separate group of 124 participants who completed the CFCS twice at a three-week interval, with no missing item responses. A statistically significant, positive and strong correlation was identified between the two administrations (r = 0.889, *p* < 0.001), and the intraclass correlation coefficient was ICC(3,1) = 0.889. Total scores did not differ significantly between administrations (paired-samples t = 1.33, *p* = 0.186), and subscale test–retest correlations ranged from 0.733 to 0.817. These findings indicate excellent temporal stability of the scale over the three-week interval (Table 11).

#### 3.3.5. Association Between the Clean Food Consumerism Scale and the Sustainable and Healthy Eating Behaviors Scale

The CFCS total score showed a very strong positive correlation with the Perceived Health Benefits subscale and strong positive correlations with the Production Process Transparency, Authenticity, Perceived Ease of Use, and Familiarity subscales (r = 0.758–0.876, all *p* < 0.001), and the subscales were moderately intercorrelated (r = 0.456–0.603, all *p* < 0.001) (Table 12).

The CFCS and SHEBS total scores showed a moderate positive correlation (r = 0.441, *p* < 0.001), and all five subscale scores were positively correlated with the SHEBS total score (r = 0.316–0.402, all *p* < 0.001) (Table 12). These positive correlations support the criterion validity of the CFCS.

## 4. Discussion

Although clean food consumerism has recently attracted increasing attention, there remains no consensus in the literature on its definition, nor are there standardized instruments for assessing consumer perceptions of clean food. The CFCS, developed by Hino and Sparks, is one of the first comprehensive instruments designed for assessing clean food consumerism across the dimensions of perceived health benefits, perceived ease of use, familiarity, authenticity, and production process transparency [7]. This study evaluated the validity and reliability of the CFCS Turkish version. The findings demonstrated that the Turkish version exhibited a high internal consistency level, preserved the original five-factor structure, and showed satisfactory psychometric properties supporting its construct validity. EFA and CFA results confirmed the validity of the theoretical structure of the scale in a Turkish sample. These findings support the CFCS as a valid and reliable tool for assessing clean food consumerism among adults in Türkiye.

In the initial stage of the Turkish validation study of the CFCS, a pilot study was conducted to assess the clarity and applicability of the scale items and identify any potential issues associated with the design of the instrument. In the pilot analysis, the overall Cronbach’s alpha was 0.950 and the alpha-if-item-deleted values ranged from 0.947 to 0.949, demonstrating high reliability [23]. Consequently, the validity analysis was performed without removing any items from the scale.

Following the reliability analysis, validity analyses were conducted in the main study. In the original scale developed by Hino and Sparks, Cronbach’s alpha coefficients for the five factors ranged from 0.79 to 0.84, whereas values between 0.83 and 0.90 were reported in the external validation conducted using a sample from the United Kingdom (UK) [7]. In the present study, the overall Cronbach’s alpha coefficient of the 18-item CFCS was 0.948, indicating high internal consistency. Although very high Cronbach’s alpha coefficients may indicate potential item redundancy, the corrected item–total correlations ranged from 0.597 to 0.757, with none exceeding the suggested threshold of 0.85 [20,23,24]. Furthermore, Cronbach’s alpha values ranged from 0.944 to 0.947 when individual items were deleted, indicating that removal of any item did not improve the internal consistency of the scale. These findings suggest that the high Cronbach’s alpha coefficient reflects strong internal consistency without evidence of substantial item redundancy. The findings are consistent with the reliability results reported for the original scale and support the reliability of the Turkish version of the CFCS. In addition, the test–retest analysis demonstrated a strong positive correlation between CFCS total scores obtained at baseline and after three weeks (r = 0.889, *p* < 0.001). This finding further supports the temporal stability of the Turkish version of the CFCS over the three-week interval.

In this study, the KMO value was 0.927, indicating a high level of sampling adequacy for factor analysis [25]. In addition, Bartlett’s test of sphericity was statistically significant (*p* < 0.001), indicating that the correlation matrix was suitable for factor analysis. Consistent with the original scale development study [7], these findings supported the appropriateness of the data for examining the factor structure of the Turkish version of the CFCS.

In the original scale development study conducted by Hino and Sparks, a five-factor structure comprising 18 items was confirmed, explaining 66.24% of the total variance [7]. In the present study, the five-factor structure of the scale was likewise retained, with the five factors collectively accounting for 80.92% of the total variance. Although the first unrotated component accounted for 53.30% of the variance, suggesting a substantial shared component among the items, the subsequent oblique (Promax) rotation, which allows correlations among factors, supported a five-factor solution. The five-factor solution was further supported by Horn’s Parallel Analysis, in which only the first five empirical factors exceeded the corresponding eigenvalues generated from random data. This multidimensional structure was further supported by the CFA, which demonstrated acceptable fit for the five-factor model in an independent sample. Standardized factor loadings ranged from 0.748 to 0.938; no item demonstrated a factor loading below the recommended threshold of 0.50 [26]. Composite reliability (0.873–0.940) and average variance extracted (0.697–0.771) supported convergent validity, and both the Fornell–Larcker criterion and HTMT ratios (all ≤ 0.69) supported discriminant validity. Furthermore, no cross-loading issues were identified in the EFA; therefore, no item was removed from the scale. The resulting 18-item, five-factor structure comprises the following dimensions: Perceived Health Benefits, Perceived Ease of Use, Familiarity, Authenticity, and Production Process Transparency. The identification of the same five dimensions as in the original scale, together with the findings from both EFA and CFA, supports the multidimensional structure of the Turkish version of the CFCS. This multidimensional structure is also consistent with the broader consumer literature, in which perceptions of food “cleanliness” are shaped not only by health and naturalness but also by processing level, ingredient familiarity, trust, and transparency regarding food production [9,27,28]. These findings reveal that clean food consumerism can be evaluated through similar dimensions across cultures and offer support for the construct validity of the Turkish adaptation.

Furthermore, the CFA findings supported the five-factor structure of the scale. In the initially specified model, the incremental indices met the recommended criteria, but the RMSEA point estimate (0.079) fell only marginally below the conventional 0.08 boundary and the upper limit of its 90% confidence interval (0.084) exceeded it, so mediocre approximate fit could not be excluded. Examination of the standardized residual correlations and modification indices located this misfit mainly in two places: the item pair 1–2 within the Perceived Health Benefits subscale, and item 12 of the Familiarity subscale, which showed elevated residuals with several items of other factors, particularly Authenticity. Items 1 and 2 are the only items in the Perceived Health Benefits subscale that express a general evaluation of the health and nutritional value of clean food, whereas the remaining four refer to naturalness and production-related attributes; this shared content plausibly generates covariation beyond the common factor. Item 12, which refers to foods eaten in childhood, touches on tradition and personal food history and therefore overlaps conceptually with the Authenticity dimension; in a food culture in which traditional and home-made foods are closely associated with naturalness, this overlap may be particularly pronounced. To preserve the structure of the original instrument, this pattern was not modelled. Allowing a single residual covariance between items 1 and 2 improved fit appreciably (RMSEA = 0.066, 90% CI 0.061–0.071; CFI = 0.965; TLI = 0.956) while leaving the loadings, composite reliability, average variance extracted and latent factor correlations essentially unchanged. The acceptable model fit observed in the CFA, obtained in a sample independent of the one used for the exploratory analysis, therefore provided additional support for the structural validity of the Turkish version of the CFCS. Nevertheless, the fit of the final model should be regarded as acceptable rather than close (RMSEA = 0.066), and because the residual covariance was specified in the same sample, Model 2 should be considered partly exploratory until it is replicated. Together with the five-factor solution identified by EFA, these findings indicate that the factor structure of the original instrument was retained in the Turkish adaptation.

The original study of the CFCS was conducted among Israeli Jewish, Israeli Arab, and UK samples and revealed that clean food consumerism represents a multidimensional construct comprising the factors of perceived health benefits, perceived ease of use, familiarity, authenticity, and production process transparency [7]. In the present study, the preservation of the five-factor structure suggests that the key components underlying clean food consumerism can be interpreted similarly across different cultures. Moreover, the fact that no items required removal during the Turkish adaptation process and that all subscales were retained indicates that the conceptual structure of the scale remains valid in the target culture.

As the CFCS and SHEBS examine common characteristics associated with health-conscious and sustainable food choices, the SHEBS was employed for assessing criterion validity. Statistically significant associations were observed between the SHEBS total score and both the CFCS subscale scores and the CFCS total score (*p* < 0.001). This finding aligns with a previous study demonstrating that healthy eating behaviors significantly impacted clean food purchasing decisions. Suvittawat and Suvittawat reported a positive association between healthy eating behaviors and clean food purchasing decisions (β = 0.440, *p* < 0.001) [29]. Therefore, the shared health-oriented characteristics of clean food consumption and sustainable healthy eating behaviors may account for the positive association noted in the present study. Moreover, this finding supports the criterion validity of the CFCS. Previous studies have reported comparable findings demonstrating the association of food choice motives with sustainability and their positive effect on healthier dietary patterns [30,31].

Food choice originates from a multifactorial etiology, including health-related concerns, sustainability issues, product characteristics, and individual behavioral tendencies [30]. Similarly, Allès et al. demonstrated that individuals who prioritize sustainability-related values in their food choices, including ethics, environmental concerns, and local production, were more likely to follow healthier dietary patterns [31]. Collectively, these findings suggest a possible common driving force behind clean food consumption and sustainable healthy eating behaviors. Furthermore, in their systematic review, Andrade and Bolini reported that clean food consumerism is conceptually closer to personal health motivations than to environmental concerns, while remaining positioned among instruments for sustainable food behavior [32]. This perspective may help explain the positive correlation observed between the CFCS and SHEBS in the present study.

This study had several limitations. First, the data were collected through an online survey, and all information was based on participants’ self-reports. In addition, participants were recruited through social media using a non-probability snowball sampling approach, which may have introduced selection and self-selection bias. Individuals with internet access or a greater interest in health and food-related issues may therefore have been more likely to participate, which should be considered when generalizing the findings to the broader Turkish adult population. Second, the majority of the sample comprised individuals with at least a high school education, potentially limiting the generalizability of the findings to populations with different educational backgrounds. Third, the final measurement model includes one residual covariance that was identified from the standardized residuals of the present sample. Although this modification was restricted to a single parameter and is supported by the shared content of the two items concerned, it is in part data-driven and requires confirmation in an independent sample. In addition, item 12 showed residual covariation with items of other factors, particularly Authenticity, which was not modelled in order to preserve the structure of the original instrument; its conceptual overlap with the Authenticity dimension should be examined in future studies. Fourth, the subsample that completed the test–retest administration obtained lower CFCS total scores than the validity sample, so the temporal stability finding should be generalized with caution. Future studies should evaluate the scale in samples with diverse sociodemographic characteristics, and should re-examine the item 1–item 2 residual covariance and the cross-factor residuals of item 12, to further strengthen the evidence regarding the psychometric properties of the Turkish version. Nevertheless, the large sample size and comprehensive validity and reliability analyses support the Turkish version of the CFCS as a psychometrically sound instrument.

## 5. Conclusions

Although the concept of clean food consumerism has recently attracted growing attention, instruments for assessing this construct from a multidimensional perspective remain limited. To the best of our knowledge, this study represents the Turkish adaptation of one of the first comprehensive instruments developed for evaluating clean food consumerism. The CFCS Turkish version demonstrated satisfactory psychometric properties, including high internal consistency and temporal stability, and preserved the original five-factor structure. The findings support the use of the scale as a valid and reliable instrument for assessing clean food consumerism among Turkish-speaking adults. Considering the heightened interest in clean food, the availability of a culturally adapted and psychometrically sound instrument may contribute to a better understanding of consumers’ perceptions and behaviors associated with clean food, supporting future research in the fields of nutrition, public health, and consumer behavior. The validated Turkish version of the CFCS is included as Supplementary Material to aid future research (Table S3).

## Figures and Tables

**Table 1:** Clean Food Consumerism Scale reliability analysis results.

Cronbach’s Alpha	*n* (Item)
0.948	18

**Table 2:** Item–Total statistics of the Clean Food Consumerism Scale.

	Scale Mean if Item Deleted	Scale Variance if Item Deleted	Corrected Item–Total Correlation	Cronbach’s Alpha if Item Deleted
I1	58.7300	177.660	0.757	0.944
I2	58.4780	179.360	0.728	0.945
I3	58.8060	177.239	0.753	0.944
I4	58.8200	177.503	0.752	0.944
I5	58.7380	177.304	0.752	0.944
I6	58.9200	177.340	0.747	0.944
I7	59.1480	179.297	0.685	0.945
I8	59.0260	179.981	0.729	0.945
I9	59.0240	180.765	0.674	0.945
I10	58.9520	181.296	0.655	0.946
I11	58.9380	181.373	0.672	0.945
I12	58.7360	181.746	0.653	0.946
I13	59.1160	182.067	0.625	0.946
I14	58.9480	181.007	0.649	0.946
I15	59.0700	181.901	0.597	0.947
I16	58.7500	181.006	0.641	0.946
I17	58.6780	180.423	0.684	0.945
I18	58.7660	180.853	0.668	0.946

**Table 3:** KMO and Bartlett’s test results.

KMO and Bartlett’s Test		
KMO measure of sampling adequacy		0.927
Bartlett’s test of sphericity	Approx. chi-square	7525.229
	df	153
	Sig.	<0.001 ***

*** *p* < 0.001.

**Table 4:** Descriptive statistics of the demographic, health, and nutritional characteristics of participants included in the EFA study.

	Males (*n* = 150)		Females (*n* = 350)		Total (*n* = 500)	
	*n*	%	*n*	%	*n*	%
Age (year) ($\bar{X}\pm SD$)	30.34 ± 11.63		31.73 ± 11.94		31.31 ± 11.85	
Marital status						
Married	28	18.7	111	31.7	139	27.8
Single	122	81.3	239	68.3	361	72.2
Education status						
Illiterate	0	0.0	2	0.6	2	0.4
Primary school	2	1.3	7	2.0	9	1.8
Middle school	3	2.0	7	2.0	10	2.0
High School	37	24.7	97	27.7	134	26.8
Associate’s degree/bachelor’s degree	89	59.3	208	59.4	297	59.4
Postgraduate education	19	12.7	29	8.3	48	9.6
Occupation						
Civil servant	13	8.7	13	3.7	26	5.2
Worker	5	3.3	6	1.7	11	2.2
Private sector employee	23	15.3	65	18.6	88	17.6
Self-employed	27	18.0	30	8.6	57	11.4
Retired	4	2.7	20	5.7	24	4.8
Student	73	48.7	162	46.3	235	47.0
Unemployed	5	3.3	54	15.4	59	11.8
Income level						
Income less than expenses	29	19.3	69	19.7	98	19.6
Income equal to expenses	54	36.0	160	45.7	214	42.8
Income greater than expenses	67	44.7	121	34.6	188	37.6
Chronic disease status						
Yes	18	12.0	60	17.1	78	15.6
No	132	88.0	290	82.9	422	84.4
Body weight (kg) ($\bar{X}\pm SD$)	76.11 ± 7.97		62.04 ± 11.20		66.26 ± 12.18	
Body height (cm) ($\bar{X}\pm SD$)	179.66 ± 7.66		164.47 ± 6.28		169.03 ± 9.68	
BMI classification						
Normal weight	128	85.3	289	82.6	417	83.4
Overweight	22	14.7	41	11.7	63	12.6
Obese	0	0.0	20	5.7	20	4.0
BMI (kg/m^2^) ($\bar{X}\pm SD$)	23.55 ± 1.64		23.01 ± 4.61		23.17 ± 3.97	

Data are presented as *n* (%) or mean ± SD. BMI, body mass index; SD, standard deviation.

**Table 5:** Dimensions of the scale obtained as a result of exploratory factor analysis.

	Perceived Health Benefits	Production Process Transparency	Authenticity	Perceived Ease of Use	Familiarity	Communalities (h^2^)
I4	**0.970**	−0.042	0.044	−0.044	−0.054	0.829
I5	**0.918**	0.029	0.033	−0.061	−0.037	0.800
I6	**0.900**	−0.113	0.023	0.096	−0.031	0.792
I3	**0.868**	0.006	0.006	−0.004	0.011	0.772
I1	**0.725**	0.048	−0.063	0.152	0.048	0.729
I2	**0.704**	0.170	−0.085	−0.037	0.126	0.699
I16	0.009	**0.920**	−0.047	−0.005	0.016	0.831
I17	0.053	**0.916**	0.019	−0.049	0.003	0.875
I18	−0.045	**0.890**	0.046	0.091	−0.044	0.825
I15	−0.004	−0.094	**0.918**	0.083	−0.007	0.840
I14	0.011	0.084	**0.913**	−0.053	0.001	0.871
I13	−0.002	0.029	**0.841**	0.039	0.017	0.779
I9	0.006	0.011	−0.019	**0.930**	−0.006	0.858
I7	0.032	−0.028	0.051	**0.869**	0.012	0.825
I8	0.023	0.057	0.047	**0.835**	0.025	0.843
I11	−0.039	0.007	−0.012	0.030	**0.929**	0.846
I10	−0.017	−0.035	−0.103	0.135	**0.906**	0.817
I12	0.086	0.011	0.196	−0.171	**0.768**	0.736
EVR	**53.302**	**8.778**	**7.123**	**6.005**	**5.714**	**-**
CEVR	**53.302**	**62.080**	**69.203**	**75.207**	**80.922**	**-**
EV	**9.594**	**1.580**	**1.282**	**1.081**	**1.029**	**-**
α	**0.936**	**0.907**	**0.896**	**0.907**	**0.862**	**-**

I, item; EVR, explained variance ratio; CEVR, cumulative explained variance ratio; EV, eigenvalue; α, Cronbach’s alpha coefficient. Bold values indicate the primary factor loading for each item.

**Table 6:** Descriptive statistics of the demographic, health, and nutritional characteristics of participants included in the CFA study.

	Males (*n* = 414)		Females (*n* = 543)		Total (*n* = 957)	
	*n*	%	*n*	%	*n*	%
Age (year) ($\bar{X}\pm SD$)	34.57 ± 13.84		31.73 ± 12.02		32.96 ± 12.91	
Marital status						
Married	168	40.6	201	37.0	369	38.6
Single	246	59.4	342	63.0	588	61.4
Education status						
Illiterate	0	0.0	1	0.2	1	0.1
Primary school	8	1.9	16	2.9	24	2.5
Middle school	16	3.9	14	2.6	30	3.1
High School	113	27.3	146	26.9	259	27.1
Associate’s degree/bachelor’s degree	200	48.3	313	57.6	513	53.6
Postgraduate education	77	18.6	53	9.8	130	13.6
Occupation						
Civil servant	31	7.5	32	5.9	63	6.6
Worker	29	7.0	11	2.0	40	4.2
Private sector employee	91	22.0	87	16.0	178	18.6
Self-employed	107	25.8	47	8.7	154	16.1
Retired	32	7.7	22	4.1	54	5.6
Student	110	26.6	221	40.7	331	34.6
Unemployed	14	3.4	123	22.7	137	14.3
Income level						
Income less than expenses	62	15.0	125	23.0	187	19.5
Income equal to expenses	148	35.7	241	44.4	389	40.6
Income greater than expenses	204	49.3	177	32.6	381	39.8
Chronic disease status						
Yes	95	22.9	129	23.8	224	23.4
No	319	77.1	414	76.2	733	76.6
Body weight (kg) ($\bar{X}\pm SD$)	88.85 ± 12.92		66.82 ± 10.62		76.35 ± 15.98	
Body height (cm) ($\bar{X}\pm SD$)	178.15 ± 7.52		164.26 ± 5.92		170.27 ± 9.58	
BMI Classification						
Normal weight	93	22.5	321	59.1	414	43.3
Overweight	217	52.4	167	30.8	384	40.1
Obese	104	25.1	55	10.1	159	16.6
BMI (kg/m^2^) ($\bar{X}\pm SD$)	27.99 ± 3.62		24.80 ± 3.93		26.18 ± 4.11	

**Table 7:** Fit statistics of the CFCS according to CFA.

Fit Statistic	Criteria	Model 1 (Initial)	Model 2 (Final)
χ^2^ (df)	-	865.76 (125)	641.39 (124)
χ^2^/df	Reported for completeness	6.93	5.17
RMSEA	RMSEA ≤ 0.08	0.079 (90% CI 0.074–0.084)	0.066 (90% CI 0.061–0.071)
NFI	0.90 ≤ NFI ≤ 1.00	0.942	0.957
CFI	0.90 ≤ CFI ≤ 1.00	0.949	0.965
TLI	0.90 ≤ TLI ≤ 1.00	0.938	0.956
SRMR	SRMR ≤ 0.08	0.045	0.047
GFI	0.85 ≤ GFI ≤ 1.00	0.896	0.926
AGFI	0.85 ≤ AGFI ≤ 1.00	0.858	0.899

Model 1 is the initially specified correlated five-factor model; Model 2 adds a residual covariance between items 1 and 2 of the Perceived Health Benefits subscale. Both χ^2^ values are significant at *p* < 0.001.

**Table 8:** Standardized factor loadings, composite reliability and average variance extracted for the final CFA model (Model 2, *n* = 957).

Factor	Item	B	SE	z	Std. Loading	CR	AVE
Perceived Health Benefits	I1	1.000	-	-	0.767	0.940	0.724
	I2	0.974	0.028	34.28	0.748		
	I3	1.089	0.038	28.73	0.848		
	I4	1.172	0.037	31.48	0.912		
	I5	1.191	0.038	31.31	0.908		
	I6	1.178	0.038	31.24	0.907		
Perceived Ease of Use	I7	1.000	-	-	0.866	0.904	0.759
	I8	0.994	0.028	35.28	0.884		
	I9	1.010	0.030	34.16	0.864		
Familiarity	I10	1.000	-	-	0.851	0.873	0.697
	I11	1.022	0.031	32.67	0.889		
	I12	0.936	0.035	26.72	0.758		
Authenticity	I13	1.000	-	-	0.818	0.894	0.738
	I14	1.089	0.033	32.76	0.908		
	I15	1.060	0.035	30.42	0.849		
Production Process Transparency	I16	1.000	-	-	0.818	0.910	0.771
	I17	1.061	0.030	35.13	0.938		
	I18	1.015	0.031	32.64	0.876		

B, unstandardized loading; SE, standard error; CR, composite reliability; AVE, average variance extracted. All loadings are significant at *p* < 0.001; loadings fixed for scaling have no standard error. CR = (Σλ)^2^/[(Σλ)^2^ + Σ(1 − λ^2^)]; AVE = Σλ^2^/k. The residual covariance between items 1 and 2 is not included in the CR formula; when it is included, CR for Perceived Health Benefits is 0.926.

**Table 9:** Discriminant validity of the Turkish CFCS (Model 2, *n* = 957). (**A**) Fornell–Larcker criterion. (**B**) Heterotrait–monotrait (HTMT) ratios.

(**A**)					
	**PHB**	**PEU**	**FAM**	**AUT**	**PPT**
Perceived Health Benefits	0.851				
Perceived Ease of Use	0.584	0.871			
Familiarity	0.530	0.658	0.835		
Authenticity	0.587	0.594	0.638	0.859	
Production Process Transparency	0.586	0.492	0.586	0.577	0.878
**(** **B** **)**					
	**PHB**	**PEU**	**FAM**	**AUT**	
Perceived Ease of Use	0.594				
Familiarity	0.592	0.671			
Authenticity	0.616	0.612	0.685		
Production Process Transparency	0.604	0.504	0.620	0.596	

(**A**) Diagonal values are the square root of AVE; off-diagonal values are latent factor correlations. The square root of AVE exceeds every corresponding correlation for all five factors. (**B**) HTMT ratios are computed from the observed item correlations and are therefore identical for Models 1 and 2. All ratios fall below the threshold of 0.85. PHB, Perceived Health Benefits; PEU, Perceived Ease of Use; FAM, Familiarity; AUT, Authenticity; PPT, Production Process Transparency.

**Table 10:** Mean and median values of the CFCS.

Subscale	*n*	$\bar{X}\pm SD$	Median (Min–Max)	Skewness	Kurtosis
Perceived Health Benefits	957	20.49 ± 6.33	22 (6–30)	−0.644	−0.188
Production Process Transparency	957	10.38 ± 3.11	11 (3–15)	−0.652	−0.119
Authenticity	957	9.55 ± 2.93	10 (3–15)	−0.249	−0.394
Perceived Ease of Use	957	9.44 ± 2.94	9 (3–15)	−0.138	−0.424
Familiarity	957	10.05 ± 2.91	10 (3–15)	−0.529	−0.020
CFCS Total	957	59.92 ± 14.71	61 (18–90)	−0.558	0.598

**Table 11:** Test–retest reliability findings of the CFCS.

	$\bar{X}\pm SD$	Median (Min-Max)	Skewness	Kurtosis	CFCS Total—Pre-Test	CFCS Total—Post-Test
CFCS Total—Pre-test	40.38 ± 12.51	43.0 (18–70)	−0.323	−0.832	1	0.889
					-	*p* < 0.001 ***
CFCS Total—Post-test	39.68 ± 12.40	41.0 (18–73)	−0.103	−0.638	r = 0.889	1
					< 0.001 ***	-

r, Pearson correlation coefficient; *** *p* < 0.001. *n* = 124; ICC(3,1) = 0.889.

**Table 12:** Correlations among the CFCS subscale scores, the CFCS total score and the SHEBS total score.

		1	2	3	4	5	6	7
Perceived Health Benefits	r	1						
	*p*							
Production Process Transparency	r	0.558	1					
	*p*	<0.001 ***						
Authenticity	r	0.564	0.535	1				
	*p*	<0.001 ***	<0.001 ***					
Perceived Ease of Use	r	0.548	0.456	0.549	1			
	*p*	<0.001 ***	<0.001 ***	<0.001 ***				
Familiarity	r	0.536	0.550	0.603	0.594	1		
	*p*	<0.001 ***	<0.001 ***	<0.001 ***	<0.001 ***			
CFCS Total	r	0.876	0.758	0.784	0.759	0.784	1	
	*p*	<0.001 ***	<0.001 ***	<0.001 ***	<0.001 ***	<0.001 ***		
SHEBS Total	r	0.341	0.316	0.364	0.375	0.402	0.441	1
	*p*	<0.001 ***	<0.001 ***	<0.001 ***	<0.001 ***	<0.001 ***	<0.001 ***	

1, Perceived Health Benefits; 2, Production Process Transparency; 3, Authenticity; 4, Perceived Ease of Use; 5, Familiarity; 6, CFCS; 7, SHEBS; r, Pearson correlation coefficient. *** *p* < 0.001.

## Data Availability

The data presented in this study are available upon request from the corresponding author.

## Supplementary Materials

The following supporting information can be downloaded at https://www.mdpi.com/article/10.3390/nu18193274/s1, Table S1: Descriptive statistics of the demographic, health, and nutritional findings of individuals. Table S2: Complete parameter estimates for the final confirmatory factor model of the Turkish Clean Food Consumerism Scale (Model 2, n = 957). Table S3: The Turkish version of the Clean Food Consumerism Scale. File S1: Complete analysis output for the Turkish adaptation of the Clean Food Consumerism Scale.

## Abbreviations

The following abbreviations are used in this manuscript:

AGFI	Adjusted Goodness-of-Fit Index
AVE	Average variance extracted
BMI	Body mass index
CFA	Confirmatory factor analysis
CFCS	Clean Food Consumerism Scale
CFI	Comparative Fit Index
CI	Confidence interval
CR	Composite reliability
EFA	Exploratory factor analysis
FAO	Food and Agriculture Organization
GFI	Goodness-of-Fit Index
HTMT	Heterotrait–monotrait ratio
ICC	Intraclass correlation coefficient
KMO	Kaiser–Meyer–Olkin
NFI	Normed Fit Index
PCA	Principal component analysis
RMSEA	Root Mean Square Error of Approximation
SD	Standard deviation
SHEBS	Sustainable and Healthy Eating Behaviors Scale
SRMR	Standardized Root Mean Square Residual
TLI	Tucker–Lewis Index
UK	United Kingdom
