# Supplementary material for: Turkish Adaptation of the Clean Food Consumerism Scale: A Validity and Reliability Study
Source: Nutrients. 2026 Oct 5;18(19):3274. doi: 10.3390/nu18193274 (PMC13666638; doi:10.3390/nu18193274)
Supplement: Supplementary file 1 [file nutrients-18-03274-s001.zip › 3_Supplementary_Table_S1.pdf]

Table S1. Descriptive statistics of the demographic, health, and nutritional findings of individuals

	Males (n = 60)		Females (n = 120)		Total (n = 180)	
	n	%	n	%	n	%
**Age (year) ($\bar{X} \pm SD$)**	28.50 ± 10.68		28.86 ± 10.47		28.74 ± 10.51	
**Marital status**						
Married	7	11.7	12	10.0	19	10.6
Single	53	88.3	108	90.0	161	89.4
**Education status**						
Illiterate	0	0.0	1	0.8	1	0.6
Primary school	0	0.0	0	0.0	0	0.0
Middle school	1	1.7	1	0.8	2	1.1
High School	29	48.3	37	30.8	66	36.7
Associate's degree/Bachelor's degree	27	45.0	73	60.8	100	55.6
Postgraduate education	3	5.0	8	6.7	11	6.1
**Occupation**						
Civil servant	3	5.0	4	3.3	7	3.9
Worker	1	1.7	1	0.8	2	1.1
Private sector employee	5	8.3	16	13.3	21	11.7
Self-employed	7	11.7	7	5.8	14	7.8
Retired	0	0.0	1	0.8	1	0.6
Student	42	70.0	81	67.5	123	68.3
Unemployed	2	3.3	10	8.3	12	6.7
**Income level**						
Income less than expenses	15	25.0	28	23.3	43	23.9
Income equal to expenses	30	50.0	48	40.0	78	43.3
Income greater than expenses	15	25.0	44	36.7	59	32.8
**Chronic disease status**						
Yes	15	25.0	19	15.8	34	18.9
No	45	75.0	101	84.2	146	81.1
**Body weight (kg) ($\bar{X} \pm SD$)**	65.07 ± 6.93		49.23 ± 4.19		54.51 ± 9.14	
**Body height (cm) ($\bar{X} \pm SD$)**	180.03 ± 6.83		165.91 ± 6.64		170.62 ± 9.45	
**BMI classification**						
Underweight	9	15.0	84	70.0	93	51.7
Normal weight	51	85.0	36	30.0	87	48.3
**BMI (kg/m^2) ($\bar{X} \pm SD$)**	20.04 ± 1.40		17.87 ± 0.87		18.59 ± 1.48	
