# Supplementary material for: Turkish Adaptation of the Clean Food Consumerism Scale: A Validity and Reliability Study
Source: Nutrients. 2026 Oct 5;18(19):3274. doi: 10.3390/nu18193274 (PMC13666638; doi:10.3390/nu18193274)
Supplement: Supplementary file 1 [file nutrients-18-03274-s001.zip › 4_Supplementary_Table_S2.pdf]

Supplementary Table S2

Complete parameter estimates for the final confirmatory factor model of the Turkish Clean Food Consumerism Scale (Model 2, n = 957)

Model: first-order measurement model with five correlated latent factors and no cross-loadings, plus a single residual covariance between items 1 and 2 of the Perceived Health Benefits subscale. Estimator: maximum likelihood. 47 free parameters, 124 degrees of freedom. The metric of each latent variable was set by fixing its first indicator loading to 1.0; parameters fixed for scaling have no standard error.

Panel A. Factor loadings

Factor	Item	B	SE	z	p	β (standardized)	Residual variance
Perceived Health Benefits	I1	1.000	—	—	—	0.767	0.412
Perceived Health Benefits	I2	0.974	0.028	34.28	<0.001	0.748	0.441
Perceived Health Benefits	I3	1.089	0.038	28.73	<0.001	0.848	0.280
Perceived Health Benefits	I4	1.172	0.037	31.48	<0.001	0.912	0.168
Perceived Health Benefits	I5	1.191	0.038	31.31	<0.001	0.908	0.175
Perceived Health Benefits	I6	1.178	0.038	31.24	<0.001	0.907	0.178
Perceived Ease of Use	I7	1.000	—	—	—	0.866	0.250
Perceived Ease of Use	I8	0.994	0.028	35.28	<0.001	0.884	0.219
Perceived Ease of Use	I9	1.010	0.030	34.16	<0.001	0.864	0.254
Familiarity	I10	1.000	—	—	—	0.851	0.275
Familiarity	I11	1.022	0.031	32.67	<0.001	0.889	0.209
Familiarity	I12	0.936	0.035	26.72	<0.001	0.758	0.426
Authenticity	I13	1.000	—	—	—	0.818	0.331
Authenticity	I14	1.089	0.033	32.76	<0.001	0.908	0.175
Authenticity	I15	1.060	0.035	30.42	<0.001	0.849	0.280
Production Process Transparency	I16	1.000	—	—	—	0.818	0.332
Production Process Transparency	I17	1.061	0.030	35.13	<0.001	0.938	0.121
Production Process Transparency	I18	1.015	0.031	32.64	<0.001	0.876	0.233

B, unstandardized loading; β, standardized loading. Residual variance computed as $1 - \beta^2$.

Panel B. Latent factor covariances and the item residual covariance

Parameter	Estimate	SE	z	p	Standardized
Authenticity ↔ Familiarity	0.518	0.037	14.02	<0.001	0.638
Authenticity ↔ Production Process Transparency	0.490	0.037	13.14	<0.001	0.577
Perceived Ease of Use ↔ Authenticity	0.486	0.036	13.54	<0.001	0.594
Perceived Ease of Use ↔ Familiarity	0.563	0.039	14.59	<0.001	0.658
Perceived Ease of Use ↔ Production Process Transparency	0.440	0.037	11.96	<0.001	0.492
Perceived Health Benefits ↔ Authenticity	0.477	0.036	13.15	<0.001	0.587
Perceived Health Benefits ↔ Familiarity	0.449	0.036	12.37	<0.001	0.530
Perceived Health Benefits ↔ Perceived Ease of Use	0.500	0.037	13.34	<0.001	0.584
Perceived Health Benefits ↔ Production Process Transparency	0.520	0.039	13.20	<0.001	0.586
Production Process Transparency ↔ Familiarity	0.520	0.039	13.36	<0.001	0.586
I1 ↔ I2 (residual covariance)	0.297	0.024	12.32	<0.001	0.484

All ten inter-factor covariances were freely estimated. The final row is the residual covariance between items 1 and 2 included in Model 2. For this row, the standardized value is the residual correlation between the two items; expressed in the metric of the observed item variances, the covariance equals 0.206.

Panel C. Item residual variances

Item	Estimate	SE	z	p	Standardized
I1	0.594	0.029	20.23	<0.001	0.412

Item	Estimate	SE	z	p	Standardized
I2	0.635	0.031	20.40	<0.001	0.441
I3	0.392	0.021	18.94	<0.001	0.280
I4	0.235	0.015	16.15	<0.001	0.168
I5	0.255	0.016	16.42	<0.001	0.175
I6	0.255	0.015	16.53	<0.001	0.178
I7	0.287	0.019	15.17	<0.001	0.250
I8	0.240	0.017	13.94	<0.001	0.219
I9	0.299	0.020	15.32	<0.001	0.254
I10	0.322	0.022	14.68	<0.001	0.275
I11	0.234	0.020	11.88	<0.001	0.209
I12	0.550	0.030	18.41	<0.001	0.426
I13	0.384	0.022	17.23	<0.001	0.331
I14	0.195	0.017	11.21	<0.001	0.175
I15	0.339	0.021	15.80	<0.001	0.280
I16	0.461	0.025	18.17	<0.001	0.332
I17	0.143	0.016	8.99	<0.001	0.121
I18	0.291	0.019	15.24	<0.001	0.233

Standardized residual variance equals $1 - \beta^2$ and represents the proportion of item variance not explained by its factor.

The complete analysis output, including fit statistics for both the initial and the final model, the standardized residual correlations used to locate the misfit, composite reliability, average variance extracted, Fornell–Larcker and HTMT results, the test–retest analysis and the criterion validity correlations, is provided in Supplementary File S1.
