# Supplementary material for: Turkish Adaptation of the Clean Food Consumerism Scale: A Validity and Reliability Study
Source: Nutrients. 2026 Oct 5;18(19):3274. doi: 10.3390/nu18193274 (PMC13666638; doi:10.3390/nu18193274)
Supplement: Supplementary file 1 [file nutrients-18-03274-s001.zip › 5_Supplementary_Table_S3_UNCHANGED.pdf]

**Table S3.** The Turkish version of the Clean Food Consumerism Scale

| **Clean Food Consumerism Scale / Temiz Gıda Tüketiciliği Ölçeği** | | | | | |
|---|---|---|---|---|---|
| | Strongly Disagree / Kesinlikle katılmıyorum | Disagree / Katılmıyorum | Neutral / Kararsızım | Agree / Katılıyorum | Strongly Agree / Kesinlikle katılıyorum |
| **Perceived health benefits / Algılanan sağlık yararları** | | | | | |
| 1. Clean food contains a lot of vitamins and minerals. / Temiz gıda çok sayıda vitamin ve mineral içerir. | | | | | |
| 2. Clean food is healthier compared to other foods. / Temiz gıda, diğer gıdalara kıyasla daha sağlıklıdır. | | | | | |
| 3. The qualities of clean food resemble those found in organic foods. / Temiz gıdanın kalitesi, organik gıdaya benzer. | | | | | |
| 4. Clean food is not genetically modified. / Temiz gıda genetik olarak değiştirilmemiştir. | | | | | |
| 5. Clean food contains no additives. / Temiz gıda katkı maddesi içermez. | | | | | |
| 6. Clean food contains natural ingredients only. / Temiz gıda yalnızca doğal içerikler barındırır. | | | | | |
| **Perceived ease of use / Algılanan kullanım kolaylığı** | | | | | |
| 7. Clean food is easy to prepare. / Temiz gıdanın hazırlanması kolaydır. | | | | | |
| 8. Clean food can be cooked very simply. / Temiz gıda çok basit şekilde pişirilebilir. | | | | | |
| 9. Clean food takes less time to prepare. / Temiz gıdanın hazırlanması daha az zaman alır. | | | | | |
| **Familiarity / Aşinalık** | | | | | |
| 10. Clean food is what I usually eat. / Temiz gıda genellikle yediğim şeydir. | | | | | |
| 11. I am very familiar with clean food. / Temiz gıdaya çok aşinayım. | | | | | |
| 12. Clean food reminds me of what I used to eat during my childhood. / Temiz gıda, çocukluğumda yediğim yiyecekleri hatırlatır. | | | | | |
| **Authenticity / Otantiklik** | | | | | |

| | | | | | |
|---|---|---|---|---|---|
| 13. Clean food is authentic. / Temiz gıda otantiktir. | | | | | |
| 14. Clean food has traditional characteristics. / Temiz gıda geleneksel özellikler taşır. | | | | | |
| 15. Clean food is associated with my ethnic background. / Temiz gıda, etnik kökenimle ilişkilidir. | | | | | |
| **Production process transparency / Üretim sürecinde şeffaflık** | | | | | |
| 16. Packaging contains extensive information regarding ingredients and the production process. / Ambalaj, içerikler ve üretim süreci hakkında kapsamlı bilgiler içerir. | | | | | |
| 17. Food that is considered clean is often linked to a high level of transparency regarding where its components originate. / Temiz gıda olarak kabul edilen yiyecekler genellikle bileşenlerinin nereden geldiğine dair yüksek düzeyde şeffaflıkla ilişkilendirilir. | | | | | |
| 18. The production process of clean food is entirely transparent. / Temiz gıdanın üretim süreci tamamen şeffaftır. | | | | | |

The Turkish version of the Clean Food Consumerism Scale (CFCS) is freely available for academic and research purposes. Researchers are kindly requested to cite the original publication when using the scale in scientific publications or other scholarly work.
