# Supplementary material for: Turkish Adaptation of the Clean Food Consumerism Scale: A Validity and Reliability Study
Source: Nutrients. 2026 Oct 5;18(19):3274. doi: 10.3390/nu18193274 (PMC13666638; doi:10.3390/nu18193274)
Supplement: Supplementary file 1 [file nutrients-18-03274-s001.zip › 6_Supplementary_File_S1_Analysis_Output.pdf]

Supplementary File S1

Complete analysis output for the Turkish adaptation of the Clean Food Consumerism Scale

```
CFCS TURKISH ADAPTATION - COMPLETE ANALYSIS OUTPUT
Source data files and sample allocation:
   Initial pool fort the pilot, EFA, and CFA samples         n = 1,637
   Pilot study                 n =   180
   Reliability / EFA sample    n =   500
   Validity / CFA sample       n =   957
   Test-retest sample          n =   124  (separate sample; two administrarions three weeks apart)

The pilot, EFA and CFA subsamples are MUTUALLY EXCLUSIVE. Verified by two
independent checks on the source files:
   questionnaire identifiers (AnketNo): pilot AND EFA = 0, pilot AND CFA = 0,
                                       EFA AND CFA = 0; union = 1,637 records
   18-item response patterns          : EFA AND CFA = 0 matching patterns
No participant contributed to more than one of these analyses, so the CFA
sample is NOT a superset of the EFA sample.
```

PILOT STUDY (n = 180)

```
   Cronbach's alpha (18 items) = 0.950
   BMI = 18.59 +/- 1.48
   BMI categories: Underweight = 93, Normal weight = 87
```

RELIABILITY SAMPLE (n = 500)

```
   Cronbach's alpha (total, 18 items) = 0.948

   Item-total statistics (Table 2)
```

Item	Mean if del	Var if del	Corr item-total	Alpha if del
I1	58.730	177.660	0.757	0.944
I2	58.478	179.360	0.728	0.945
I3	58.806	177.239	0.753	0.944
I4	58.820	177.503	0.752	0.944
I5	58.738	177.304	0.752	0.944
I6	58.920	177.340	0.747	0.944
I7	59.148	179.297	0.685	0.945
I8	59.026	179.981	0.729	0.945
I9	59.024	180.765	0.674	0.945
I10	58.952	181.296	0.655	0.946
I11	58.938	181.373	0.672	0.945
I12	58.736	181.746	0.653	0.946
I13	59.116	182.067	0.625	0.946
I14	58.948	181.007	0.649	0.946
I15	59.070	181.901	0.597	0.947
I16	58.750	181.006	0.641	0.946
I17	58.678	180.423	0.684	0.945
I18	58.766	180.853	0.668	0.946

```
   Range of corrected item-total correlations: 0.597 - 0.757
```

EXPLORATORY FACTOR ANALYSIS (n = 500)

```
KMO = 0.927
Bartlett's test of sphericity: chi2 = 7525.229, df = 153, p < 0.001
Initial eigenvalues (PCA):
   Component 1:   9.594   %var = 53.302   cumulative =  53.302
   Component 2:   1.580   %var =  8.778   cumulative =  62.080
   Component 3:   1.282   %var =  7.123   cumulative =  69.203
   Component 4:   1.081   %var =  6.005   cumulative =  75.207
   Component 5:   1.029   %var =  5.714   cumulative =  80.922
   Component 6:   0.582   %var =  3.231   cumulative =  84.153
Five components retained (all eigenvalues > 1.0), explaining 80.922% of total variance.
Subscale Cronbach's alpha:
   Perceived Health Benefits        0.936
   Perceived Ease of Use            0.907
   Familiarity                      0.862
   Authenticity                     0.896
   Production Process Transparency  0.907
```

CONFIRMATORY FACTOR ANALYSIS (n = 957)

```
Model 1 (initial): first-order model, five correlated latent factors,
no cross-loadings, no correlated residuals. 46 free parameters, df = 125.

Model 1 fit indices
   chi2       = 865.759  (df = 125, p < 0.001)
   chi2/df    = 6.926
   CFI        = 0.949
   TLI        = 0.938
   NFI        = 0.942
   RMSEA      = 0.079  90% CI [0.074, 0.084]
   SRMR       = 0.045
   GFI        = 0.896   [Joreskog-Sorbom formula]
   AGFI       = 0.858
   Note: the RMSEA point estimate is only marginally below 0.08 and the
   upper limit of its 90% confidence interval exceeds that boundary, so
   local sources of misfit were examined.

Largest standardized residual correlations for Model 1 (top 10)
Item pair       residual r       z   factors
I1 - I2              0.164    5.07   same factor (PHB)
I1 - I12             0.160    4.95   PHB - FAM
I12 - I2             0.157    4.85   FAM - PHB
I12 - I14            0.130    4.02   FAM - AUT
I12 - I13            0.116    3.59   FAM - AUT
I12 - I15            0.110    3.41   FAM - AUT
I12 - I3             0.108    3.35   FAM - PHB
I1 - I10             0.090    2.79   PHB - FAM
I12 - I5             0.088    2.74   FAM - PHB
I13 - I7             0.086    2.65   AUT - PEU
```

The I1-I2 residual is the largest single residual. Item 12 is involved in 7 of the 10 largest residuals (with I1, I2, I3, I5 and all three AUT items). Items 1 and 2 are the only PHB items giving a general evaluation of health/ nutritional value; items 3-6 refer to naturalness and production attributes. A single residual covariance (I1 ~~ I2) was therefore added. Item 12 residuals were not modelled, to preserve the simple structure of the original scale.

Model 1 parameter estimates (initial model, for comparison with Model 2)

Factor	Item	B	SE	z	beta	resid
Perceived Health Benefits	I1	1.000	--	--	0.794	0.370
Perceived Health Benefits	I2	0.976	0.036	26.87	0.776	0.399
Perceived Health Benefits	I3	1.067	0.035	30.90	0.860	0.261
Perceived Health Benefits	I4	1.124	0.034	33.27	0.905	0.181
Perceived Health Benefits	I5	1.140	0.035	33.00	0.900	0.190
Perceived Health Benefits	I6	1.126	0.034	32.84	0.897	0.195
Perceived Ease of Use	I7	1.000	--	--	0.866	0.250
Perceived Ease of Use	I8	0.995	0.028	35.28	0.884	0.219
Perceived Ease of Use	I9	1.010	0.030	34.16	0.864	0.254
Familiarity	I10	1.000	--	--	0.851	0.275
Familiarity	I11	1.022	0.031	32.67	0.889	0.209
Familiarity	I12	0.936	0.035	26.74	0.758	0.425
Authenticity	I13	1.000	--	--	0.818	0.331
Authenticity	I14	1.089	0.033	32.76	0.908	0.175
Authenticity	I15	1.060	0.035	30.43	0.849	0.279
Production Process Transparency	I16	1.000	--	--	0.818	0.331
Production Process Transparency	I17	1.061	0.030	35.15	0.938	0.120
Production Process Transparency	I18	1.014	0.031	32.63	0.875	0.234

Standardized loading range: 0.758 - 0.938 (all p < 0.001)

Composite reliability and average variance extracted (Model 1)

Factor	k	CR	AVE	sqrtAVE
Perceived Health Benefits	6	0.943	0.734	0.857
Perceived Ease of Use	3	0.904	0.759	0.871
Familiarity	3	0.873	0.697	0.835
Authenticity	3	0.894	0.738	0.859
Production Process Transparency	3	0.910	0.771	0.878

Latent factor correlations (Model 1)

	PHB	PEU	FAM	AUT	PPT
PHB	1.000	0.589	0.542	0.595	0.590
PEU	0.589	1.000	0.659	0.594	0.492
FAM	0.542	0.659	1.000	0.639	0.586
AUT	0.595	0.594	0.639	1.000	0.576
PPT	0.590	0.492	0.586	0.576	1.000

Modification indices for Model 1 (score test, expected information; 15 largest)

Parameter	MI	EPC
I1 ~~ I2	212.11	0.287
AUT =~ I12	91.78	0.432
I2 ~~ I6	72.90	-0.134
I10 ~~ I11	70.80	0.233
I5 ~~ I6	68.95	0.108

```
I1 ~~ I3                     66.81     0.136
PHB =~ I12                   54.33     0.263
FAM =~ I1                    50.32     0.249
I2 ~~ I3                     45.98     0.116
I10 ~~ I12                   45.96    -0.156
I1 ~~ I5                     45.68    -0.104
I3 ~~ I5                     42.66    -0.089
I1 ~~ I4                     41.90    -0.096
I4 ~~ I5                     40.94     0.082
I1 ~~ I6                     39.09    -0.096
~~ residual covariance; =~ cross-loading; EPC, expected parameter change
(unstandardized). For I1 ~~ I2 the likelihood-ratio test gives delta chi2 = 224.37.

Model 2 (final): Model 1 plus a residual covariance between items 1 and 2.
47 free parameters, df = 124.

Model 2 fit indices (Table 7)
   chi2       = 641.393  (df = 124, p < 0.001)
   chi2/df    = 5.173
   CFI        = 0.965
   TLI        = 0.956
   NFI        = 0.957
   RMSEA      = 0.066  90% CI [0.061, 0.071]
   SRMR       = 0.047
   GFI        = 0.926
   AGFI       = 0.899

Model comparison: delta chi2 = 224.37, delta df = 1, p < 0.001
The upper limit of the RMSEA confidence interval now falls below 0.08.
Note on SRMR: SRMR rose from 0.0450 to 0.0470 although a parameter was added.
ML minimizes the likelihood discrepancy, not the squared residuals summarized
by SRMR. Freeing I1 ~~ I2 removed that residual but lowered the loadings of
I1 and I2, which enlarged their residuals with other items (e.g. I1-I12
0.160 -> 0.178, I2-I12 0.157 -> 0.175, I1-I3 0.071 -> 0.102), so the sum of
squared standardized residuals increased (0.347 -> 0.378). Both values were
reproduced by re-estimating the models from the raw data.

Model 2 parameter estimates (Table 8 / Supplementary Table S2)
Factor                         Item          B      SE        z    beta   resid
Perceived Health Benefits      I1        1.000   --      --      0.767   0.412
Perceived Health Benefits      I2        0.974   0.028    34.28   0.748   0.441
Perceived Health Benefits      I3        1.089   0.038    28.73   0.848   0.280
Perceived Health Benefits      I4        1.172   0.037    31.48   0.912   0.168
Perceived Health Benefits      I5        1.191   0.038    31.31   0.908   0.175
Perceived Health Benefits      I6        1.178   0.038    31.24   0.907   0.178
Perceived Ease of Use          I7        1.000   --      --      0.866   0.250
Perceived Ease of Use          I8        0.994   0.028    35.28   0.884   0.219
Perceived Ease of Use          I9        1.010   0.030    34.16   0.864   0.254
Familiarity                    I10       1.000   --      --      0.851   0.275
Familiarity                    I11       1.022   0.031    32.67   0.889   0.209
Familiarity                    I12       0.936   0.035    26.72   0.758   0.426
Authenticity                   I13       1.000   --      --      0.818   0.331
```

Authenticity	I14	1.089	0.033	32.76	0.908	0.175
Authenticity	I15	1.060	0.035	30.42	0.849	0.280
Production Process Transparency	I16	1.000	--	--	0.818	0.332
Production Process Transparency	I17	1.061	0.030	35.13	0.938	0.121
Production Process Transparency	I18	1.015	0.031	32.64	0.876	0.233

Standardized loading range: 0.748 - 0.938 (all p < 0.001)

Residual covariance I1 ~~ I2: estimate = 0.297, SE = 0.024, residual correlation = 0.484 (0.206 in observed-SD metric), p < 0.001

Composite reliability and average variance extracted (Model 2) (Table 8)

Factor	k	CR	AVE	sqrtAVE	alpha
Perceived Health Benefits	6	0.940	0.724	0.851	0.943
Perceived Ease of Use	3	0.904	0.759	0.871	0.904
Familiarity	3	0.873	0.697	0.835	0.864
Authenticity	3	0.894	0.738	0.859	0.891
Production Process Transparency	3	0.910	0.771	0.878	0.905

Note: CR excludes the I1 ~~ I2 residual covariance; including it gives CR(PHB) = 0.926.

Latent factor correlations (Model 2)

	PHB	PEU	FAM	AUT	PPT
PHB	1.000	0.584	0.530	0.587	0.586
PEU	0.584	1.000	0.658	0.594	0.492
FAM	0.530	0.658	1.000	0.638	0.586
AUT	0.587	0.594	0.638	1.000	0.577
PPT	0.586	0.492	0.586	0.577	1.000

Discriminant validity - Fornell-Larcker (diagonal = sqrt(AVE)) (Table 9)

	PHB	PEU	FAM	AUT	PPT
PHB	0.851	0.584	0.530	0.587	0.586
PEU	0.584	0.871	0.658	0.594	0.492
FAM	0.530	0.658	0.835	0.638	0.586
AUT	0.587	0.594	0.638	0.859	0.577
PPT	0.586	0.492	0.586	0.577	0.878

Violations: none - discriminant validity supported

Discriminant validity - HTMT ratios (computed from observed correlations; identical for Models 1 and 2)

Perceived Health Benefits	- Perceived Ease of Use	0.594
Perceived Health Benefits	- Familiarity	0.592
Perceived Health Benefits	- Authenticity	0.616
Perceived Health Benefits	- Production Process Transparency	0.604
Perceived Ease of Use	- Familiarity	0.671
Perceived Ease of Use	- Authenticity	0.612
Perceived Ease of Use	- Production Process Transparency	0.504
Familiarity	- Authenticity	0.685
Familiarity	- Production Process Transparency	0.620
Authenticity	- Production Process Transparency	0.596

Maximum HTMT = 0.685 (threshold 0.85)

Modification indices for Model 2 (score test, expected information; 15 largest)

Parameter	MI	EPC
AUT =~ I12	91.89	0.432
I10 ~~ I11	69.78	0.232

```
PHB =~ I12                    53.40     0.266
I10 ~~ I12                    45.59    -0.156
I1 ~~ I3                      44.91     0.101
FAM =~ I1                     38.16     0.191
I3 ~~ I5                      37.83    -0.085
PPT =~ I12                    37.49     0.230
PEU =~ I13                    34.14     0.202
I12 ~~ I14                    34.09     0.087
AUT =~ I11                    32.96    -0.232
I14 ~~ I15                    32.57     0.145
I5 ~~ I6                      29.85     0.072
PEU =~ I14                    26.85    -0.169
PEU =~ I1                     26.44     0.165
After I1 ~~ I2 is freed, the largest remaining indices concern item 12,
chiefly its cross-loading on Authenticity; these were not modelled.
```

DESCRIPTIVE STATISTICS (n = 957, Table 10)

```
Perceived Health Benefits         20.49 +/-  6.33  median 22 (6-30)  skew -0.644  kurtosis -0.188
Perceived Ease of Use              9.44 +/-  2.94  median 9 (3-15)  skew -0.138  kurtosis -0.424
Familiarity                       10.05 +/-  2.91  median 10 (3-15)  skew -0.529  kurtosis -0.020
Authenticity                       9.55 +/-  2.93  median 10 (3-15)  skew -0.249  kurtosis -0.394
Production Process Transparency   10.38 +/-  3.11  median 11 (3-15)  skew -0.652  kurtosis -0.119
CFCS TOTAL                        59.92 +/- 14.71  median 61 (18-90)  skew -0.558  kurtosis  0.598
```

CRITERION VALIDITY (n = 957, Table 12)

```
Pearson correlations (all p < 0.001):
   Perceived Health Benefits        vs SHEBS Total  r = 0.341   (Spearman rho = 0.322)
   Perceived Ease of Use            vs SHEBS Total  r = 0.375   (Spearman rho = 0.358)
   Familiarity                      vs SHEBS Total  r = 0.402   (Spearman rho = 0.384)
   Authenticity                     vs SHEBS Total  r = 0.364   (Spearman rho = 0.348)
   Production Process Transparency  vs SHEBS Total  r = 0.316   (Spearman rho = 0.302)
   CFCS Total                       vs SHEBS Total  r = 0.441   (Spearman rho = 0.429)
CFCS total vs subscales:
   Perceived Health Benefits        r = 0.876
   Perceived Ease of Use            r = 0.759
   Familiarity                      r = 0.784
   Authenticity                     r = 0.784
   Production Process Transparency  r = 0.758
```

TEST-RETEST RELIABILITY (three-week interval, n = 124, Table 11)

```
Missing item responses: 0
Pre-test  total: 40.38 +/- 12.51  median 43.0 (18-70)  skew -0.323  kurtosis -0.832
Post-test total: 39.68 +/- 12.40  median 41.0 (18-73)  skew -0.103  kurtosis -0.638
Pearson r = 0.889 (p < 0.001)   Spearman rho = 0.863
ICC(3,1) = 0.889
Paired-samples t = 1.329, p = 0.1863 (no significant change)
Subscale test-retest correlations:
```

Perceived Health Benefits	r = 0.817
Perceived Ease of Use	r = 0.773
Familiarity	r = 0.762
Authenticity	r = 0.748
Production Process Transparency	r = 0.733

Note: subscale columns D5/D5.1 in the source workbook contain an off-by-one row reference; all scores were recomputed from the item columns.
